# Combined inhibition of PD-1/PD-L1, Lag-3, and Tim-3 axes augments antitumor immunity in gastric cancer–T cell coculture models

**DOI:** 10.1007/s10120-020-01151-8

**Published:** 2021-02-20

**Authors:** Kosaku Mimura, Ley-Fang Kua, Jin-Fen Xiao, Bernadette Reyna Asuncion, Yuko Nakayama, Nicholas Syn, Zul Fazreen, Richie Soong, Koji Kono, Wei-Peng Yong

**Affiliations:** 1 Department of Gastrointestinal Tract Surgery, Fukushima Medical University School of Medicine, Fukushima, Japan; 2 Department of Blood Transfusion and Transplantation Immunology, Fukushima Medical University School of Medicine, Fukushima, Japan; 3 Department of Haematology-Oncology, National University Health System (S) Pte Ltd, Level 7, NUHS Tower Block, 1E Kent Ridge Road, Singapore, 119228 Singapore; 4 Cancer Science Institute, National University of Singapore, Singapore, Singapore; 5 Division of Hematology/Oncology, Cedar-Sinai Medical Center, ULCA School of Medicine, Los Angeles, CA USA; 6 First Department of Surgery, University of Yamanashi, Yamanashi, Japan; 7 Department of Pathology, National University of Singapore, Singapore, Singapore

**Keywords:** Gastric cancer, Combinatorial immunotherapy, PD-1, Tim-3, Lag-3

## Abstract

**Background:**

Immunotherapy targeting PD-1 provides a limited survival benefit in patients with unresectable advanced or recurrent gastric cancer (GC). Beside PD-L1, the expression of inhibitory ligands such as CEACAM-1 and LSECtin on GC cells account for this limitation. Here we assessed their expression and immune suppressive effect in GC patients.

**Methods:**

Using multiplexed immunohistochemistry staining, we evaluated the distribution of different inhibitory ligands, including PD-L1, CEACAM-1, LSECtin, and MHC class II, in 365 GC patients. We analyzed their correlations and overall survival (OS) based on the expression of each inhibitory ligand and the independent prognostic factors that affect OS. Subsequently, we evaluated the additive effect of anti-PD-1 mAb or anti-PD-L1 mAb with/without anti-Lag-3 mAb with/without anti-Tim-3 mAb in cytotoxic assay using tumor-antigen specific CTL clones against GC cell lines.

**Results:**

Co-expression of the inhibitory ligands for PD-1, Tim-3, and Lag-3 was observed in the largest proportion (34.7%). CEACAM-1, LSECtin, and MHC class II expression showed significant correlation with PD-L1 expression and OS. Multivariable analysis demonstrated that CEACAM-1 low is an independent prognostic factor. Furthermore, combining dual and triple ICIs yielded additive effect on cytotoxicity of CTL clones against each immune inhibitory ligand positive GC cell lines.

**Conclusions:**

Our findings suggested that the expression of inhibitory ligands for Tim-3 and Lag-3 on GC cells serve as potential biomarkers to predict the response to anti-PD-1 therapy and the combinatorial immunotherapy with ICIs targeting for PD-1, Tim-3, and Lag-3 has a therapeutic potential for GC patients.

**Supplementary information:**

The online version contains supplementary material available at (10.1007/s10120-020-01151-8).

## Background

Gastric cancer (GC) was the second leading cause of cancer-related deaths and the sixth most prevalent malignant diseases worldwide in the GLOBOCAN 2018 database [1]. For patients with advanced or metastatic GC, standard-of-care treatment options include platinum compounds plus fluoropyrimidines, docetaxel, paclitaxel or irinotecan, as well as anti-VEGFR2 monoclonal antibody (mAb), ramucirumab, alone or in combination with paclitaxel [2–7]. Immune checkpoint blockage targeting programmed cell death 1 (PD-1) on T cell such as nivolumab and pembrolizumab has been emerging, and remains as backbone in GC immunotherapy and serve as a promising treatment strategy for advanced GC patients. In ATTRACTION-2 study, patients with unresectable advanced or recurrent gastric or gastroesophageal junction (GEJ) cancer irresponsive to conventional therapy were treated with anti-PD-1 mAb, nivolumab, and an overall response rate (ORR) of 11.2% was achieved [8]. Meanwhile, KEYNOTE-059/Cohort 1 patients with locally advanced or metastatic gastric or GEJ adenocarcinoma treated with pembrolizumab also yielded an ORR of 11.6% [9]. These observations suggest that immune checkpoint blockage with anti-PD-1 mAb is beneficial for advanced GC patients. However, ORR of advanced GC patients toward anti-PD-1 mAb remains to be modest and exploring novel treatment strategies such as combinatorial therapies to overcome resistance to cancer immunotherapy are necessary to improve the ORR.

Co-expression of PD-1 and Lymphocyte activation gene-3 (Lag-3), or PD-1 and T cell immunoglobulin-3 (Tim-3) facilitated T cell exhaustion and led to tumoral immune escape [10–14]. Therefore, besides combining anti-PD-1 mAb with radiotherapy or chemotherapy, a rational approach to improve the efficacy of anti-PD-1 mAb also include combining anti-PD-1 mAb with other immune checkpoint inhibitors (ICIs), such as anti-Lag-3 mAb and anti-Tim-3 mAb. Currently, anti-PD-1 mAb in combination with ICI targeting Lag-3 and Tim-3 are being explored in clinical trials such as NCT01968109, NCT03662659 and AMBER study (NCT02817633). For combinatorial therapy using anti-PD-1 mAb with anti-Lag-3 mAb or anti-Tim-3 mAb to work effectively, the presence of CTLs in the tumor microenvironment is essential. Many types of CD8 positive T cells present in the tumor microenvironment, including CTLs and exhausted T cells, etc. However, only activated T cells such as CTLs in CD8 positive T cell populations attack tumor cells. A CD8 T effector gene signature including *CD8A*, *CD8B*, *EOMES*, *GZMA*, *GZMB*, *IFNG*, and *PRF1* was established to identify activated T cells and can be used to evaluate the abundance of CTLs in the tumor microenvironment [15]. Higher expression levels of CD8 T effector gene signature indicate more activated CD8 positive T cells in the tumor, which has been reported to have prognostic effect in patient survival [16]. However, it was also observed that ultimate survival outcome varies although a high CD8 T effector gene signature was observed [17]. The expression of T cell inhibitory ligands, including programmed death ligand-1 (PD-L1), PD-L2, carcinoembryonic antigen-related adhesion molecule-1 (CEACAM-1), lymph node sinusoidal endothelial cell C-type lectin (LSECtin), and major histocompatibility complex (MHC) class II, on cancer cells also affects the anti-tumor activity of activated CD8 positive T cells such as CTLs. There have been some reports showing evidences regarding the expression levels of these ligands in GC patients [18–20].

In the present study, we sought to explore a new strategy for advanced or recurrent GC patients using anti-PD-1 mAb in combination with anti-Tim-3 mAb and/or anti-Lag-3 mAb. We evaluated the correlation between the number of tumor infiltrating CD8 positive T cells and expression levels of inhibitory immune checkpoint ligands, including ligand for PD-1 (PD-L1), ligand for Tim-3 (CEACAM-1), and ligands for Lag-3 (LSECtin and MHC class II) on tumor cells, and the correlation between inhibitory ligands by interrogating TCGA stomach adenocarcinoma tissue dataset and by performing the multiplex immunohistochemistry (IHC) staining in surgically resected GC samples. Finally, we also evaluated the additive effect of anti-PD-1 mAb or anti-PD-L1 mAb with/without anti-Tim-3 mAb with/without anti-Lag-3 mAb using tumor-specific CTL clones.

## Methods

### Clinical samples

Samples from 365 patients for tissue arrays of intestinal and diffuse primary gastric tumors were collected. These patients underwent surgery for GC in National University Hospital of Singapore between 2000 and 2013. It was approved by the Domain Specific Review Board of the National Healthcare Group of Singapore (Reference 2015/00,209). All GC staging was performed according to the latest American Joint Committee on Cancer (8th edition).

### Multiplex IHC staining

Multiplex IHC staining was performed using Leica Bond RX machine (Leica Biosystems, Wetzlar, Germany) with Opal Multiplex fIHC kit (Perkin Elmer, Waltham, MA). Three micrometer thickness of tissue microarray sections were stained with primary antibodies against the following: CEACAM-1 (clone EPR4048, dilution 1:100; abcam, Cambridge, UK), LSECtin (clone EPR13724, dilution 1:250; abcam), MHC class II (clone EPR11226, dilution 1:2500; abcam), PD-L1 (clone SP263, dilution 1:5; Ventana Medical Systems, Tucson, AZ), CD8 (clone 4B11, dilution 1:3000; Novocastra, Newcastle upon Tyne, UK), and Cytokeratin (clone AE1/AE3, dilution 1:100; abcam). Followed by secondary antibodies, fluorophore (FITC, Cy3, Cy5, Texas Red)-conjugated tyramides signal amplification buffer (Perkin Elmer) were added. After six sequential reactions, sections were counterstained with DAPI and mounted with Vectashield fluorescence mounting medium (Vector Labs, Burlingame, CA). Human tonsil formalin-fixed, paraffin-embedded tissues stained with or without primary antibody were used as positive and negative controls in each IHC staining.

IHC stains were examined by a pathologist (BGRA) using the Vectra digital slide imaging system and the Inform software (Perkin Elmer, Waltham, MA). Individual cells were graded for tumor (PD-L1, CEACAM-1, LSECtin, MHC class II, and Cytokeratin) staining on membranous compartments. A histoscore was generated by adding the products of the proportion at each intensity grading level of 0, + 1, + 2, and + 3 with a maximum score of 300. The density of CD8 positive immune shaped cells was generated and expression of CD8 was grouped in high and low by using the median, as a cut-off. Protein expression was generated from an average histoscore and density from three representative images at 20 × magnification was presented.

### Tumor proportion score (TPS) of each inhibitory ligand

Expression of each inhibitory ligand was assessed by the TPS. TPS ≥ 10% was used for PD-L1 and standardized at ≥ 25% for MHC class II (25% used in [21]), CEACAM-1 (20–30% used in [22, 23]) and LSECtin (no report on TPS).

### TCGA dataset analysis

To elucidate the clinical relevance of inhibitory ligands of interest, the correlations of mRNA expression levels of PD-L1 (*CD274*), CEACAM-1 (*CEACAM1*), LSECtin (*CLEC4G*), MHC class II (*HLA-DRA*), and CD8 T effector gene signature including *CD8A, CD8B, EOMES, GZMA, GZMB, IFNG, and PRF1* in stomach adenocarcinoma tissues was analyzed [15]. The mRNA expression z-scores of genes (RNA Seq V2 RSEM) were retrieved from TCGA stomach adenocarcinoma tissue (PanCancer Atlas, *n* = 401) dataset through cBioPortal (http://www.cbioportal.org/) [19, 20].

### Tumor cell lines

Kinesin family member 20A (KIF20A)-expressing gastric adenocarcinoma-derived cell line, MKN45, was used in the present study, according to our previous observation that human leukocyte antigen (HLA)-A24 restricted, KIF20A specific CTL clone from patient recognized MKN45 [24, 25]. MKN7 and NUGC3, HLA-A24-restricted and KIF20A expressing cell lines, were included in cytotoxic assay for further functional evaluation [26, 27]. HLA-A02 restricted and KIF20A expressing gastric adenocarcinoma-derived cell line, OE19, was used as negative control [27, 28]. All cell lines were purchased from American Type Culture Collection (Manassas, VA). The authenticity of cell lines was confirmed by short tandem repeat profiling. Cell lines were cultured in RPMI-1640 containing L-Glutamine (Invitrogen, Carlsbad, CA) supplemented with 5% FBS (Invitrogen) and 1% penicillin–streptomycin (Sigma Aldrich, St.Louis, MO).

### Cell treatment and reagents

ICIs used in the present study: anti-PD-L1 mAb (H12), anti-PD-1 mAb (B6co), anti-PD-1 mAb (Nivolumab), anti-Tim-3 mAb and anti-Lag-3 mAb, were kindly provided by Dr. Cheng-I Wang (Singapore Immunology Network, Astars, Singapore). CTL clones or target cell lines were treated with 1 µg/mL mAb for 1 h before coculture experiments. In ELISpot and cytotoxic assays, target cell lines were treated with anti-PD-L1 mAb (H12), while CTL clones were treated with anti-Tim-3 mAb, anti-Lag-3 mAb, anti-PD-1 mAb (B6co) or anti-PD-1 mAb (Nivolumab).

### Generation of HLA-A24 restricted, KIF20A peptide-specific CTL clone

The HLA-A24 restricted, KIF20A peptide-specific CTL clones were established using HLA-A24 positive peripheral blood mononuclear cells (PBMC) from healthy donors and advanced GC patients as previously described [26]. PBMC from advanced GC patients who were enrolled in clinical trial (ClinicalTrials.gov Identifier: NCT01227772) were highly immunogenic to KIF20A peptide [24]. Briefly, T cells were stimulated with KIF20A peptide-loaded, mitomycin C treated (Kyowa Hakko Kirin, Tokyo, Japan) autologous mature dendritic cells every 7 days. Cultures were maintained in AIM-V containing L-Glutamine (Invitrogen), supplemented with 10% pooled human AB serum (Invitrogen) and 1% penicillin–streptomycin (Sigma Aldrich). After the third stimulation on day 21, the CTL lines were tested for their antigen specificity for the KIF20A peptide using ELISpot assay. A peptide-specific CTL clone was established from an HLA-A24 restricted, KIF20A peptide-specific CTL line by using a limiting dilution method.

### ELISpot assay

The antigen specific CTL response was determined by interferon-γ (IFN-γ) ELISpot assay according to the manufacturer’s protocol (Mabtech, Stockholm, Sweden). Briefly, 96-well plates with nitrocellulose membranes (Millipore, Bedford, MA) were pre-coated with primary anti-IFN-*γ* antibody (1-D1K) at 4 °C overnight. The plates were blocked with AIM-V medium containing 5% human serum (Invitrogen). Target cells (2 × 10^4^/well) and CTL clones (2 × 10^3^/well) were co-cultured in 200 µL of culture medium for 24 h in triplicate. These wells were treated with biotinylated secondary anti-IFN-*γ* antibody (7-B6-1), followed by incubation with HRP-reagent and stained with TMB (Mabtech). The spots were then quantified with automated ELISpot reader, ImmunoSPOT S4 (Cellular Technology Ltd, Cleveland, OH). Positive CTL responding specifically to the vaccinated peptide were evaluated and classified according to a previously described algorithm [29].

### Cytotoxic assay

Cytotoxic activity of the specific CTL clones was measured by a calcein-release assay as previously described [26]. Briefly, target cells (MKN45, NUGC3, and MKN7) were stained with 5 µM of calcein-AM (DOJINDO LABORATORIES, Kumamoto, Japan) for 30 min in cell culture incubator. Stained targets (5 × 10^3^ cells/well) were co-cultured with different ratios of effector cells, CTL clones, in culture medium (200 µL) for 4 h. Assays were performed in triplicate in 96-well U-bottomed plate. After incubation, 100 µL of the supernatant was collected and the fluorescence in the supernatant was measured using Infinite 200 (Tecan Group Ltd., Männedorf, Switzerland). Spontaneous release was obtained from target cells incubated without effector cells, and maximum release was obtained from detergent-lysed target cells. The percentage of specific lysis was calculated according to the following formula: % specific lysis = 100 × (experimental release—spontaneous release)/(maximum release—spontaneous release).

### Flow cytometry

MKN7, MKN45, and NUGC3 cell lines were incubated with fluorochrome-conjugated mAb for 30 min at 4 °C. Antibodies used: PE-conjugated anti-human PD-L1 (CD274; B7-H1) (clone MIH1; eBioscience, Santa Clara, CA), PE-conjugated anti-human CEACAM-1 (CD66a) (Biolegend, San Diego, CA), APC-conjugated anti-human Galectin-9 (Biolegend), FITC-conjugated anti-human LSECtin (Santa Cruz, Dallas, TX), APC-conjugated anti-human HLA-DR (Biolegend). Staining was evaluated by LSRII flow cytometer (BD Biosciences, Franklin Lakes, NJ).

### Statistics

Association between clinicopathological parameters and inhibitory ligands and CD8 expression was evaluated by Chi-square, Fisher’s exact test or Mann–Whitney U test. Two-tailed paired Student’s *t*-test was performed to compare two groups. Comparison of multiple groups was performed with Anova with Tukey's post hoc test. Overall survival (OS) was displayed with Kaplan–Meier plot and calculated with logrank p test. Cox proportional hazard regression model was used to identify independent risk factors that affect patient’s OS. Correlation analysis was performed with Pearson’s correlation and significance was calculated with Fisher’s exact test. All error bars represent mean ± SEM. *p* values less than 0.05 were considered statistically significant. Statistical analyses were performed using the GraphPad Prism software (GraphPad Software Inc., San Diego, CA) and SPSS (IBM SPSS Statistics for Windows, version 26.0; IBM Corp., Armonk, NY).

## Results

### Association of immune inhibitory ligands and CD8 expression with clinicopathological characteristics

**Fig. 1:**
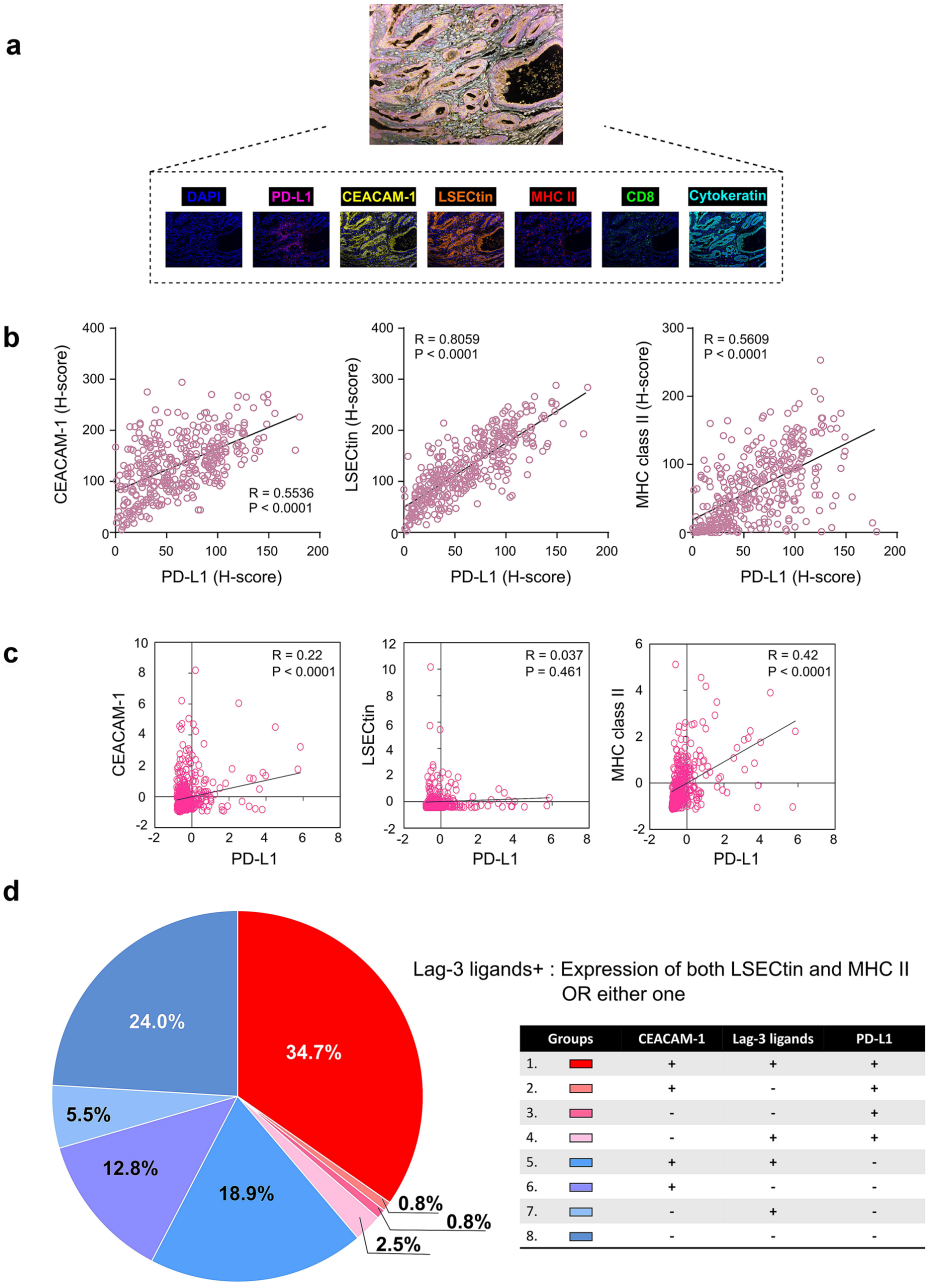
Correlation and distribution of PD-L1, CEACAM-1, LSECtin, and MHC class II expression in GC. **a** Representative IHC staining at 20 × magnification. Upper: Merge. Lower: DAPI (opal), PD-L1 (magenta), CEACAM-1 (yellow), LSECtin (orange), MHC class II (red), CD8 (green), and Cytokeratin (cyan). The correlation between PD-L1 and each inhibitory ligand expression in the H-score of IHC (**b**) and between mRNA expression of PD-L1 and that of CEACAM-1 or LSECtin or MHC class II in the TCGA stomach adenocarcinoma tissue dataset (**c**). **d** Pie chart representing the proportion of patients with respective expression of inhibitory ligands, based on the TPS ≥ 10% for PD-L1 and ≥ 25% for CEACAM-1, LSECtin, and MHC class II. Lag-3 ligands + : expression of both LSECtin and MHC class II or either one

The clinicopathological characteristics of 365 patients with GC, comprising 252 men and 113 women as described in Table 1. These clinical samples were then subjected to multiplex IHC staining to evaluate the expression levels of each inhibitory ligand (PD-L1, CEACAM-1, LSECtin, and MHC class II), cytotoxic T cell marker CD8 and tumor marker Cytokeratin (Fig. 1a). High CD8 infiltration was observed in diffuse and mixed type in Lauren’s Classification (*p* = 0.014), stage I (*p* = 0.011) and T1/2 (*p* = 0.001). PD-L1 expression was higher in the M0 group (*p* = 0.035). High CEACAM-1 expression was observed more in well differentiated than in other histological subtypes (*p* = 0.05). High LSECtin expression was observed more in intestinal type (*p* = 0.053). High MHC class II expression was observed more in patients with higher mean age of 67.7 (*p* = 0.035) and more in intestinal type (*p* = 0.011).

**Table 1:**
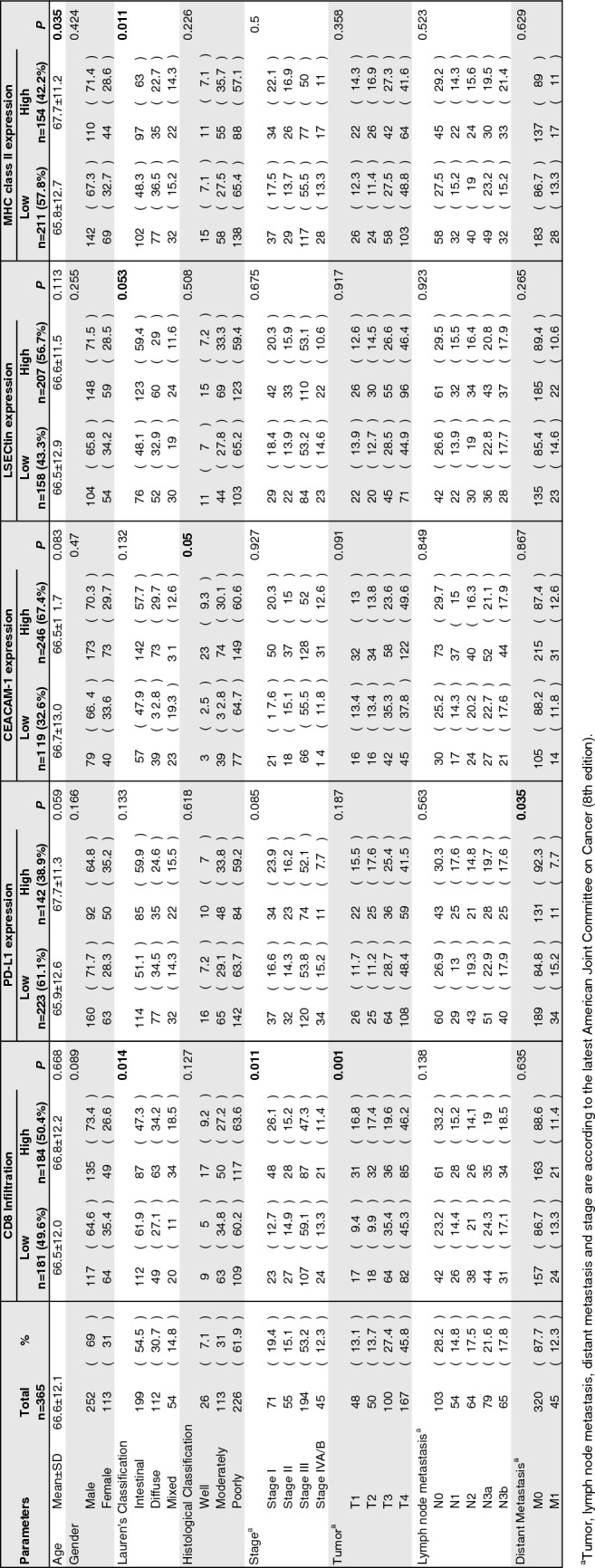
Association of ligands and CD8 expression with clinicopathological characteristics of gastric cancer patients by immunohistochemistry

### Co-expression of immune inhibitory ligands in GC patients

H-score of CEACAM-1 (ligand for Tim-3) or LSECtin (ligand for Lag-3) or MHC class II (ligand for Lag-3) were positively correlated with H-score of PD-L1 (ligand for PD-1), suggesting these inhibitory ligands may co-express in GC (Fig. 1b). A positively correlation was also noted between mRNA expression of CEACAM-1 or MHC class II and that of PD-L1 in TCGA stomach adenocarcinoma tissue dataset (*r* = 0.22 and *p* < 0.0001, *r* = 0.42 and *p* < 0.0001 respectively) (Fig. 1c). Based on the TPS ≥ 10%, 142 patients (38.8%) had PD-L1-positive tumors in IHC staining, which is consistent with previous studies [30–34]. The distribution of patients expressing different combination of inhibitory ligands in 365 GC patients was presented in pie chart (Fig. 1d). There were 8 different combination groups and among the PD-L1 positive groups, 34.7% expressed the ligands for PD-1, Tim-3, and Lag-3, called as “triple positive” group, which made up the largest proportion of patients, followed by the second larger proportion (24.04%) as “triple negative” group (Fig. 1d).

### Expression level of immune inhibitory ligands correlated with the frequency of CD8 positive T cells

Next, the correlation between the H-score of inhibitory ligands and the frequency of CD8 positive T cells was explored. A significant correlation was observed between the frequency of CD8 positive T cells and the H-score of PD-L1, CEACAM-1, LSECtin, and MHC class II in our GC patient cohort (Supplementary Fig. S1a). This was in concordance with the observation in TCGA stomach adenocarcinoma tissue dataset analysis, showing a significant correlation between CD8 T effector gene signature and mRNA expression levels of PD-L1, CEACAM-1, and MHC class II, but not LSECtin (Supplementary Fig. S1b).

### Clinical significance of each inhibitory ligand expression in GC

**Fig. 2:**
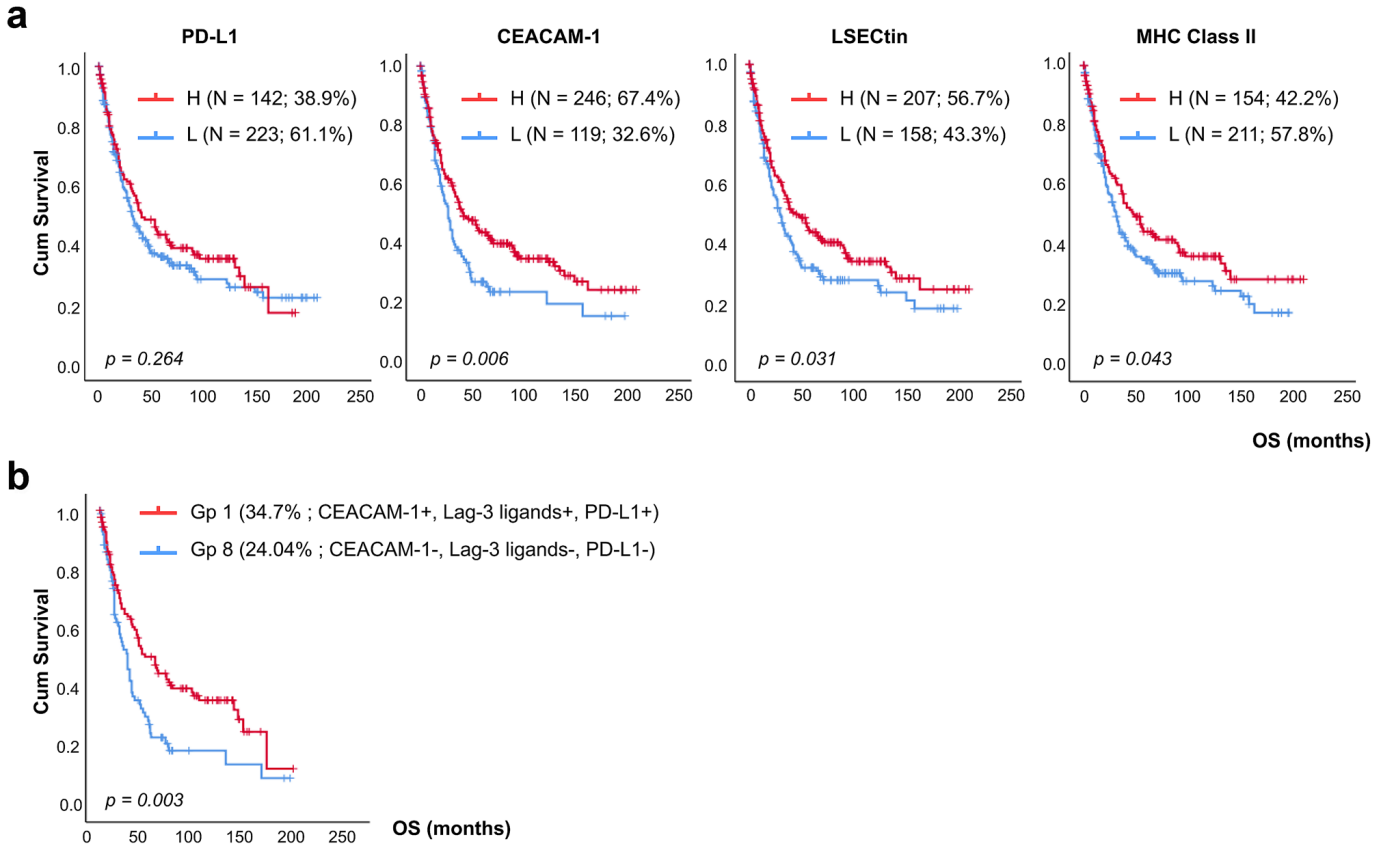
Kaplan–Meier curves for OS in patients with GC. **a** OS stratified by PD-L1, CEACAM-1, LSECtin, and MHC class II expression status. **b** Kaplan–Meier curve analysis between “triple-positive” and “triple-negative” group of patients as mentioned in Fig. 1d

To evaluate the prognostic value of expression of each inhibitory ligand in GC patients, patients’ survival was analyzed according to the status of PD-L1, CEACAM-1, LSECtin, and MHC class II. Patients with high expression of the ligands trended toward a better survival, except for the PD-L1 (Fig. 2a). Subsequently, the OS between the triple positive group and various combination groups more than 10% defined in Fig. 1d was analyzed. The triple positive group had a significantly better OS than triple negative group but not to other groups (Fig. 2b and Supplementary Fig. S2). Factors such as low CEACAM-1, low LSECtin, low MHC class II, older age, advanced staging, diffuse type, and undifferentiated histology were associated with poor OS according to COX univariable proportional hazards analysis (Table 2). Further, low CEACAM-1 status, besides older age, advanced staging, and diffuse type, was also associated with poor OS in multivariable analysis (HR = 1.512, *p* = 0.003) (Table 2).

**Table 2:** Univariable and multivariable Cox regression analysis of OS among GC patients

Variables	HR	95% CI	*p* value	HR	95% CI	*p* value
Gender	0.93	0.70–1.23	0.562			
(male vs. female)						
PD-L1	1.17	0.89–1.53	0.268			
(high vs. low)						
CEACAM-1	1.459	1.11–1.92	0.007	1.512	1.15–2.00	0.003
(high vs low)						
LSECtin	1.331	1.02–1.73	0.033			
(high vs. low)						
MHC class II	1.317	1.01–1.72	0.045			
(high vs. low)						
Age	1.836	1.40–2.41	< 0.0001	1.99	1.51–2.62	< 0.0001
(< 68 vs. > 68)						
Stage	2.405	1.79–3.24	< 0.0001	2.381	1.76–3.23	< 0.0001
(I-II vs. III-IV)						
Lauren	1.396	1.07–1.82	0.013	1.308	1.00–1.71	0.049
(Intestinal vs. diffuse)						
Histology	1.377	1.04–1.83	0.026			
(Differ vs. undiffer)						

### Additive effect of combinatorial ICIs therapy in anti-tumor CTL activity

With the observation of co-expression of inhibitory ligands on tumor cells, we hypothesized that dual or triple blocking for the inhibitory ligands could enhance the cytotoxicity of CTLs against GC cells. Since we used HLA-A24 restricted, KIF20A peptide-specific CTL clones, KIF20A-expressing HLA-A2402-positive GC cell lines including MKN45, MKN7, and NUGC3 were selected for subsequent ex-vivo experiments as targets [24, 25, 27]. On the other hand, OE19 was used as negative control since it is KIF20A expressing HLA-A24 negative GC cell line [26–28]. The expression of inhibitory ligands on GC cell lines was evaluated by flow cytometry using antibodies specific for the PD-L1, CEACAM-1, Galectin-9, LSECtin, and MHC class II. Subsequently, cytotoxic assay was performed using CTL clones with ICIs to evaluate the effect of ICIs in the single, dual, and triple setting.

**Fig. 3:**
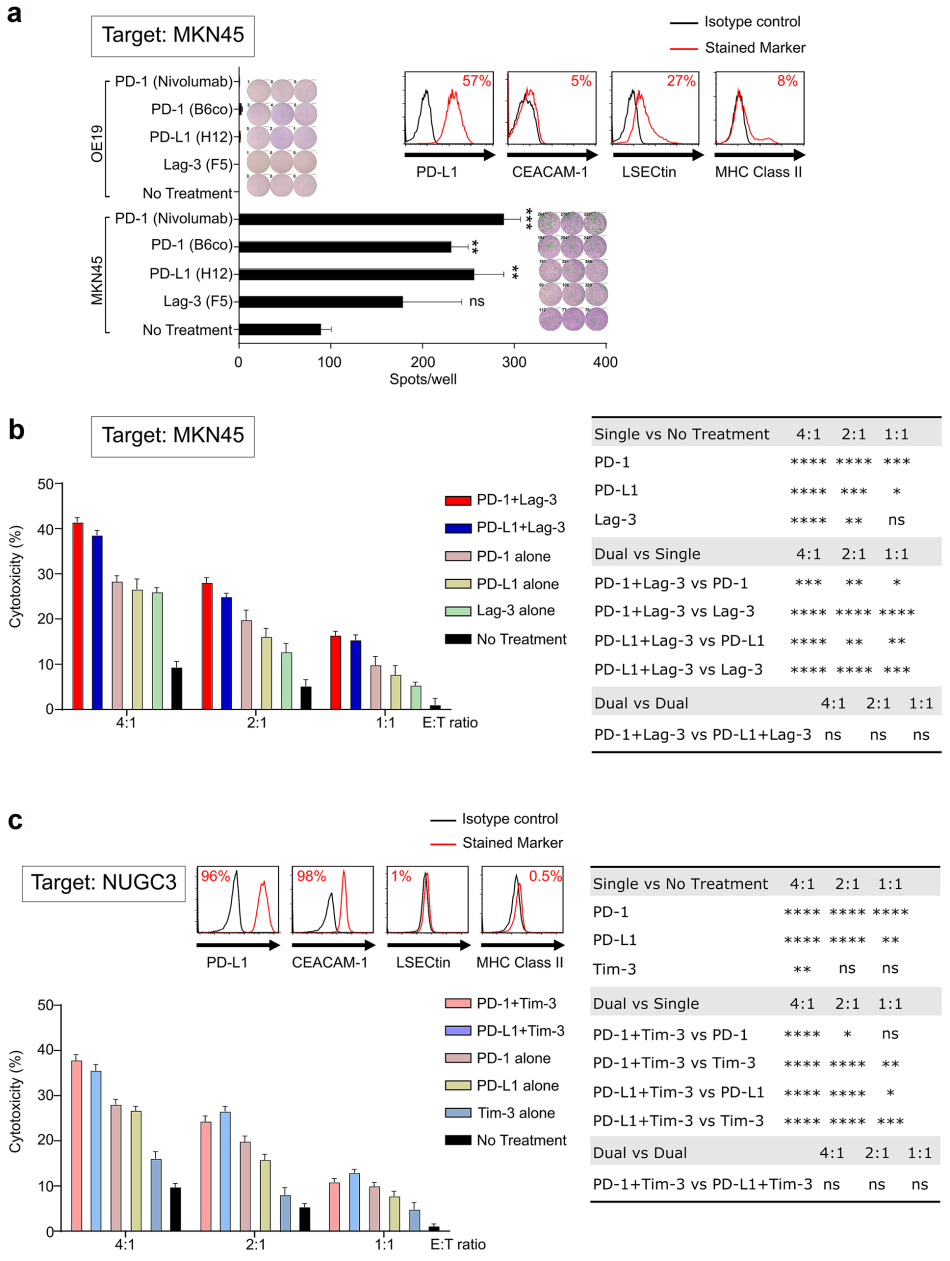
Effect of one ICI and two different types of ICIs against tumor cells. **a** MKN45 was assessed for PD-L1, CEACAM-1, LSECtin, and MHC class II expression by flow cytometry. Representative histograms are shown on the upper right. Response of CTL clones treated with/without ICIs against MKN45 and OE19 treated with/without anti-PD-L1 mAb was assessed by quantifying the amount of IFN-*γ* produced from CTL clones using ELISpot assay. Number of IFN-γ producing cells was quantified (left, with bar chart; right, with representative photomicrographs of wells). **b** Cytotoxic activity of CTL clones were assessed in single and dual therapy setting; CTL clones treated with/without anti-PD-1 mAb or/plus anti-Lag-3 mAb against MKN45 treated with/without anti-PD-L1 mAb. **c** NUGC3 was assessed for PD-L1, CEACAM-1, LSECtin, and MHC class II expression by flow cytometry. Representative histograms of each inhibitory ligand staining are shown (upper). Cytotoxicity assessment using CTL clones treated with/without anti-PD-1 or/plus anti-Tim-3 mAb against NUGC3 treated with/without anti-PD-L1 mAb. Comparison between combinations were analyzed as shown on the right. E:T ratio, effector:target ratio. Each bar was performed in triplicate. *p < 0.05, **p < 0.01, ***p < 0.001, ****p < 0.0001, ns (not significant) by two-way ANOVA and Tukey’s test

Since MKN45 expressed PD-L1 (ligand for PD-1) and LSECtin (ligand for Lag-3) (Fig. 3a upper right), the monotherapy of ICIs using anti-PD-1 mAb, anti-PD-L1 mAb, and anti-Lag-3 mAb was evaluated by ELISpot assay and cytotoxic assay. In ELISpot assay using MKN45, up-regulation of IFN-*γ* production from CTL clones was observed when CTL clones were reactivated with anti-PD-1 or anti-Lag-3 mAb, or tumor cells treated with anti-PD-L1 mAb (Fig. 3a). On the other hand, expectedly, no production of IFN-*γ* from CTL clones was observed when OE19 was used as target (Fig. 3a). A significantly higher cytotoxicity of CTL clones against MKN45 was observed in the single treatment setting using anti-PD-1 or anti-Lag-3, or anti-PD-L1 mAb as compared with no treatment setting (Fig. 3b). Furthermore, in dual combinatorial setting, anti-PD-1 mAb plus anti-Lag-3 mAb or anti-PD-L1 mAb plus anti-Lag-3 mAb also led to significant additive effects compared with single treatment setting, no significant difference was observed between these two dual combinatorial setting (Fig. 3b). Even though MKN45 did not express CEACAM-1, a significantly higher cytotoxicity in single setting of anti-Tim-3 compared with no treatment and some significant additive effects in dual combinatorial setting of anti-Tim-3 mAb plus anti-PD-1 mAb or anti-PD-L1 mAb or anti-Lag-3 mAb compared with single treatment setting were also observed in MKN45, because MKN45 expressed Galectin-9 that is an another ligand for Tim-3 (Supplementary Figure. S3).

To evaluate the effect of ICIs on CTL cytotoxicity for NUGC3, which expressed PD-L1 and CEACAM-1, but not LSECtin and MHC class II (ligand for Lag-3) (Fig. 3c upper), CTL clones and NUGC3 were subjected to ICIs using anti-PD-1 mAb, anti-PD-L1 mAb, and anti-Tim-3 mAb in single and dual treatment setting. Single anti-Tim-3 mAb treatment did not exert significant increase in cytotoxicity compared with no treatment, except in a higher ratio at 4:1, while single anti-PD-1 or anti-PD-L1 mAb exert significant increase in cytotoxicity in all ratio compared with no treatment (Fig. 3c). In contrast, dual combination exerted a significant degree of additive effect compared with no treatment (*p* < 0.0001 at all ratio, asterisks not shown). Significant additive cytotoxicity was also observed in dual combination of anti-PD-1 mAb plus anti-Tim-3 mAb or anti-PD-L1 mAb plus anti-Tim-3 mAb compared with single treatment, whereas no significant difference observed between these two dual combinatorial setting (Fig. 3c). In concordance with the lack of ligand expression for Lag-3, no cytotoxicity in single setting or additive effect was observed from anti-Lag-3 mAb treatment (Supplementary Figure. S4).

**Fig. 4:**
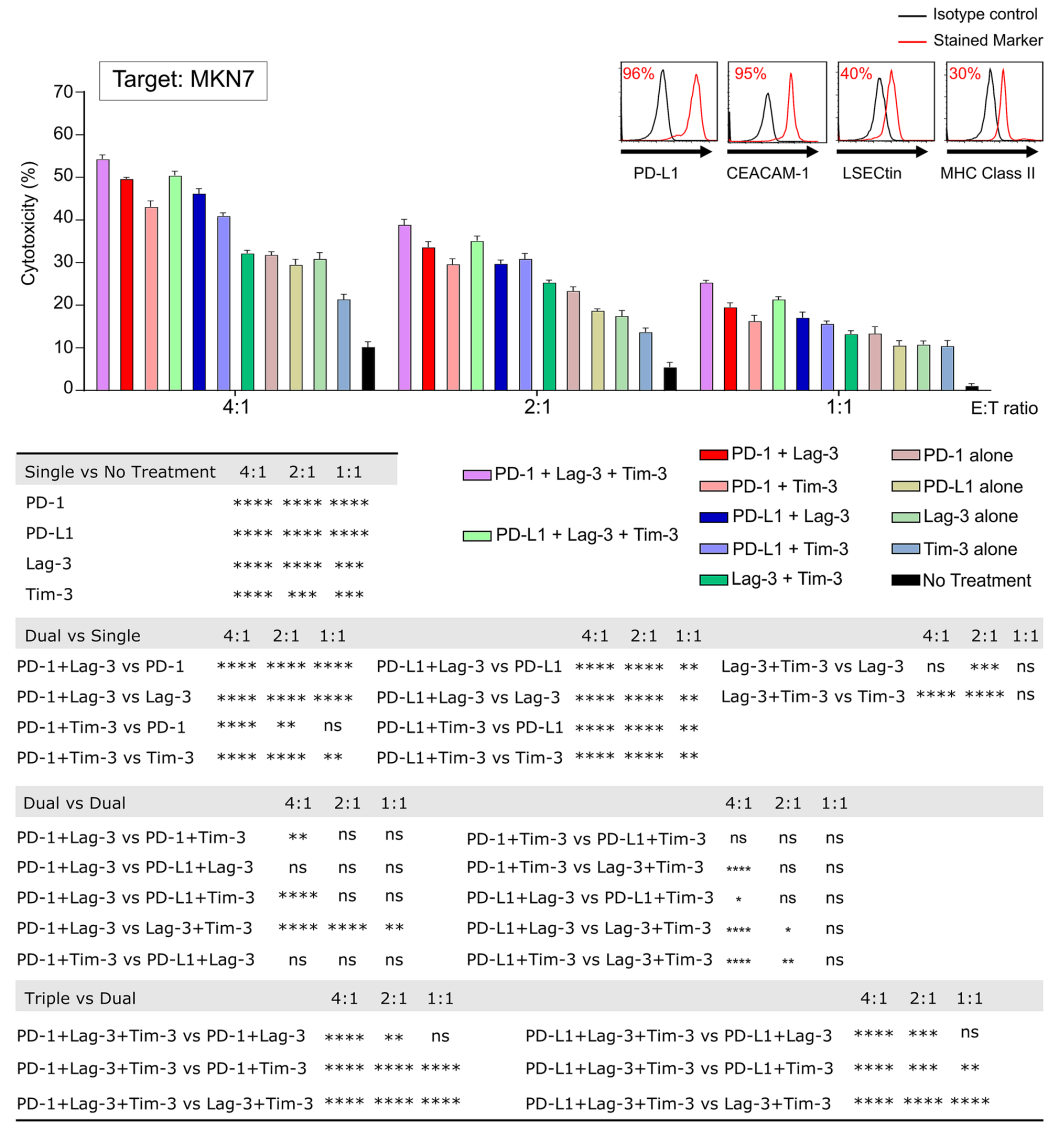
Additive effect of three different types of ICIs against tumor cells. MKN7 was assessed for PD-L1, CEACAM-1, LSECtin, and MHC class II expression by flow cytometry. Representative histograms of each inhibitory ligand staining are shown (upper right). Cytotoxicity assessment using CTL clones treated with/without anti-PD-1 or/plus anti-Tim-3 or/plus anti-Lag-3 mAb against MKN7 treated with/without anti-PD-L1 mAb. Comparison between combinations were analyzed as shown on the bottom. E:T ratio, effector:target ratio. Each bar was performed in triplicate. *p < 0.05, **p < 0.01, ***p < 0.001, ****p < 0.0001, ns by two-way ANOVA and Tukey’s test

As the triple positive group was the largest proportion in our GC patient cohort (Fig. 1d), we hypothesized that this population might benefit from the triple blockade therapy. In the cytotoxic assay using MKN7, which expressed PD-L1, CEACAM-1, LSECtin, and MHC class II (Fig. 4 top right), the dual combination exerted a significant degree of additive cytotoxicity compared with no treatment (*p* < 0.0001 at all ratio, asterisks not shown) and with single treatment where mostly *p* < 0.0001 at both ratio 4:1 and 2:1, except for the combination of anti-Lag-3 plus anti-Tim-3 mAb versus single anti-Lag-3 mAb (Fig. 4). Notably, a significantly higher anti-tumor CTL activity was facilitated in the triple combination of ICIs compared with dual combinations (Fig. 4).

### Discussion

Limited subset of GC patients at advanced or metastatic stages benefit from immunotherapy targeting PD-1 axis [8, 9]. It was therefore suggested that the efficacy of anti-PD-1 mAb was limited by the presence of other co-inhibitory receptors, such as Tim-3 and Lag-3 [35]. In the present study, we demonstrated that ligands for inhibitory receptors, such as PD-L1, CEACAM-1, LSECtin, and MHC class II, significantly co-expressed on GC cells and their low expression group had a poor OS except for the PD-L1, and low CEACAM-1 status retained its prognostic significance in multivariable analysis. Monotherapy of anti-PD-1 mAb, anti-PD-L1 mAb, anti-Tim-3 mAb, and anti-Lag-3 mAb led to a significantly higher cytotoxicity of tumor antigen specific CTL clones against GC cells as compared to no treatment control. Notably, the combination therapy of anti-PD-1 mAb or anti-PD-L1 mAb plus anti-Tim-3 mAb plus/or anti-Lag-3 mAb showed remarkably additive effect in the cytotoxicity of the tumor antigen specific CTL clones against GC cells as compared to ICI monotherapy.

Multiple co-inhibitory receptors, such as CTLA-4, PD-1, Tim-3, and Lag-3, have emerged as significant inhibitory factors in CTL responses. Among these targets, Tim-3 and Lag-3 show considerably high potential because their co-expression with PD-1 have been reported to dysfunction tumor-specific T cells [10–14]. Koyama S et al. also reported that up-regulation of Tim-3 and Lag-3 in patients with metastatic lung cancer developed adaptive resistance to PD-1 inhibitors [36]. Furthermore, Tim-3 inhibitor was reported to exhibit similar efficacy as PD-1 inhibitor [37], and Lag-3 inhibitor also showed synergistic effect with PD-1 inhibitor [11]. In the present study, we also focused on PD-1, Tim-3, and Lag-3 axes because CEACAM-1 (ligand for Tim-3) was highly expressed in gastric adenocarcinoma tissues but not in normal gastric mucosa [18], and gastroesophageal tumor samples had higher expression of LSECtin (ligand for Lag-3) than corresponding adjacent normal tissues [19], and expression of MHC class II (ligand for Lag-3) on solid tumor cells was found to have negative impact by promoting T cell anergy [20].

T cell exhaustion caused by inhibitory immune checkpoints remains as a highlighted knotty issue in the area of immunotherapy. Although both PD-1 signaling and Lag-3 signaling in CTLs inhibit the T cell receptor (TCR) signaling [38–44], each immune checkpoint inhibitor responses through different mechanisms uniquely [45]. PD-1 signaling in T cells recruits SHP2 and SHP2 induces the attenuation of TCR signaling by dephosphorylating the CD3 chain, resulting in downregulation of IFN-*γ* production [38–41]. Lag-3 signaling provides negative regulation of TCR signaling and KIEELE domain is required for the function of Lag-3 [38, 42–44]. As preclinical relevancies, it was noted that the blockade of PD-1 and Lag-3 signaling restored IFN-*γ* secretion of CD8 T cells in mice and human model [11, 14, 46, 47]. In line with these reports, our results showed that CTL clones with PD-1 or Lag-3 signaling blockade using their inhibitors can produce IFN-γ more than the untreated group in ELISpot assay (Fig. 3a).

Co-expression of multiple inhibitory receptors is a prevailing key hallmark of exhausted CD8 positive T cells observed in both chronic infection and cancer settings, and the PD-1/PD-L1 axes has been the main focus in the inhibitory receptor pathway involved in T cell exhaustion. In the event of restoring the function of CTL using ICIs co-blockade of PD-1 and Lag-3 had showed synergistic reversal of T cell exhaustion, and similar co-blockade effects was demonstrated in other combinations of inhibitory receptors [48–51]. In view of this, the evaluation of their ligand expression, such as CEACAM-1 for Tim-3 and LSECtin for Lag-3, on tumor cells is important to prove the inhibition by their axis in the tumor microenvironment. In addition, it was reported that inhibiting their ligands are ideal strategies to display similar phenomenon as anti-PD-1 mAb and to exert synergistic effect in enhancing antitumor functions [52]. In line with these reports, our results showed that PD-L1 expression correlated significantly with CEACAM-1, LSECtin, and MHC class II expression on GC cells (Fig. 1b). These results encouraged us to have the combination therapy using anti-PD-1 mAb or anti-PD-L1 mAb plus anti-Tim-3 mAb or anti-Lag-3 mAb for GC patients.

In fact, ICI targeting Tim-3 or Lag-3 are progressing in Phase I clinical trial, such as the monotherapy with anti-Lag-3 mAb in patients with advanced solid tumor malignancies or lymphomas (ClinicalTrials.gov Identifier: NCT03489369), and the combination therapy using anti-Lag-3 mAb and anti-PD-1 mAb in patients with recurrent glioblastoma (ClinicalTrials.gov Identifier: NCT02658981) and using anti-Tim-3 mAb and anti-PD-1 mAb in patients with advanced solid tumors (ClinicalTrials.gov Identifier: NCT02817633). In addition, many underlying mechanisms including de-novo and acquired resistance [53] have been indeed accountable for the tumor immune escape activities, and in fact, many other combinations with ICIs have been performed and showed promising outcome, such as chemoimmunotherapy combination in triple-negative breast cancer [54] and radioimmunotherapy combination in indolent lymphoma [55]. Notably, a group from Philadelphia performed “a triple blow for cancer” whereby combination of anti-CTLA-4 Ab and radiotherapy with anti-PD-L1 Ab have demonstrated the effectiveness in impeding tumor immune escape through distinct mechanisms [56]. In the future, direct analysis of immunogenic status in tumor microenvironment including multiple immune checkpoint molecules may be required to optimize adjuvant immunotherapeutic strategies for each patient.

In conclusion, our results indicated that Tim-3 and Lag-3 as well as PD-1 axis suppress the immune response in the tumor microenvironment of GC. With an aim to improve clinical benefits in a wider population of GC patients, our findings suggested that the expression of ligands for Tim-3 and Lag-3 on tumor cells serve as the potential biomarker to detect the candidates for anti-PD-1 therapy in GC patients, and the combinatorial immunotherapy with ICIs targeting for PD-1, Tim-3, and Lag-3 has a therapeutic potential in GC patients.

## Supplementary information

Supplementary file1 (PDF 2698kb)

Supplementary file2 (PDF 1540kb)

Supplementary file3 (PDF 7362kb)

Supplementary file4 (PDF 7562kb)
