# Supplementary material for: Combined inhibition of PD-1/PD-L1, Lag-3, and Tim-3 axes augments antitumor immunity in gastric cancer–T cell coculture models
Source: Gastric Cancer. 2021 Feb 20;24(3):611–23. doi: 10.1007/s10120-020-01151-8 (PMC8065004; doi:10.1007/s10120-020-01151-8)
Supplement: Supplementary file 1 — Supplementary file1 (PDF 2698kb) [file 10120_2020_1151_MOESM1_ESM.pdf]

Supplementary Figure S1

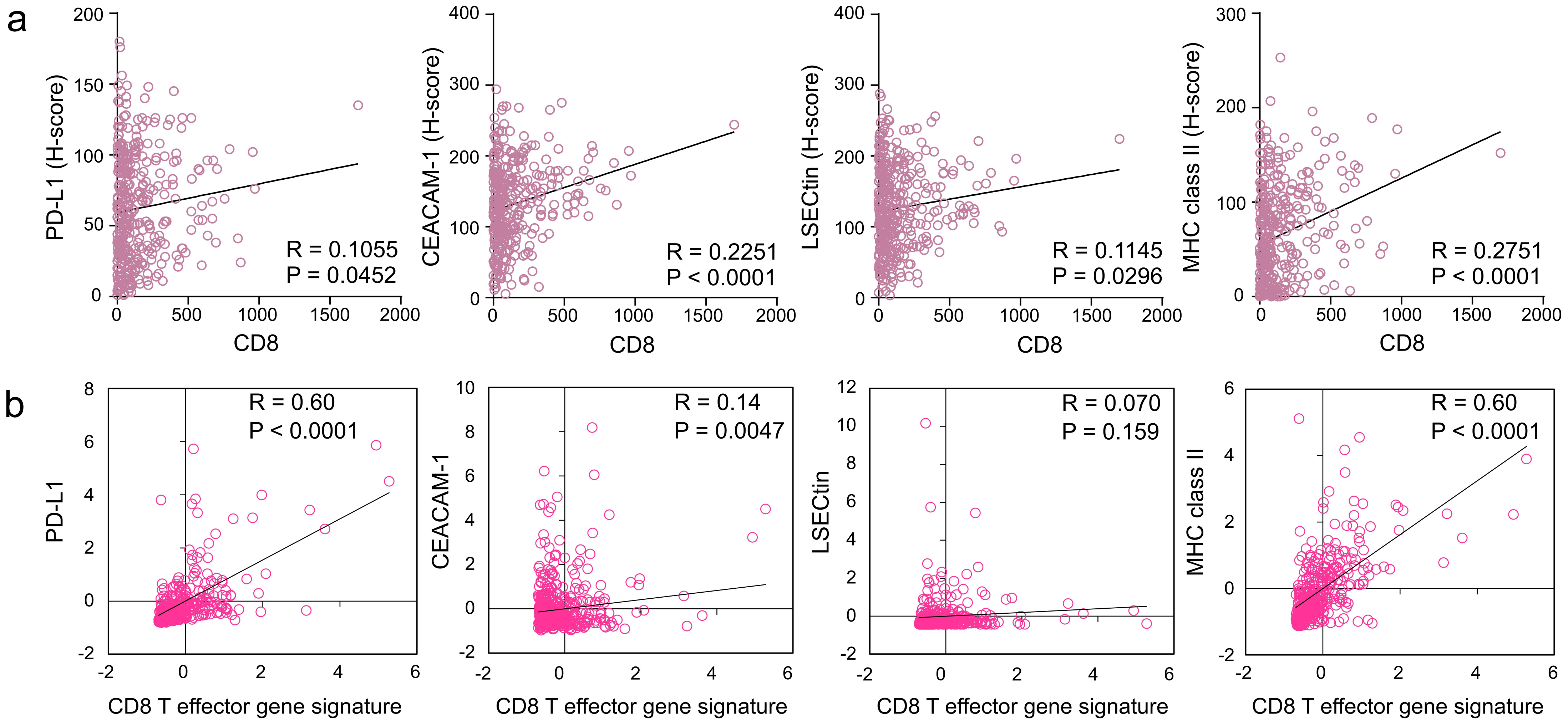

Supplementary Figure S1. The correlation of immune suppressive ligands and CD8 positive T cells

The correlation between the H-score of each inhibitory ligand and the number of CD8 positive T cells in IHC staining of GC tissues (**a**) and between CD8 T effector gene signature and mRNA expression of PD-L1, CEACAM-1, LSECtin, and MHC class II respectively in TCGA GC dataset (**b**).
