# Supplementary material for: Combined inhibition of PD-1/PD-L1, Lag-3, and Tim-3 axes augments antitumor immunity in gastric cancer–T cell coculture models
Source: Gastric Cancer. 2021 Feb 20;24(3):611–23. doi: 10.1007/s10120-020-01151-8 (PMC8065004; doi:10.1007/s10120-020-01151-8)
Supplement: Supplementary file 2 — Supplementary file2 (PDF 1540kb) [file 10120_2020_1151_MOESM2_ESM.pdf]

Supplementary Figure S2

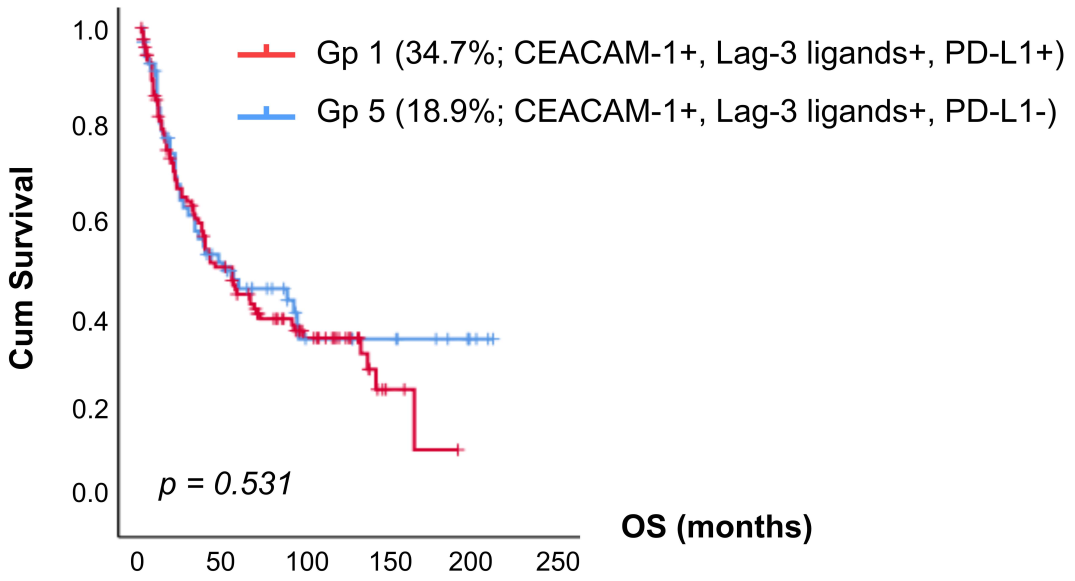

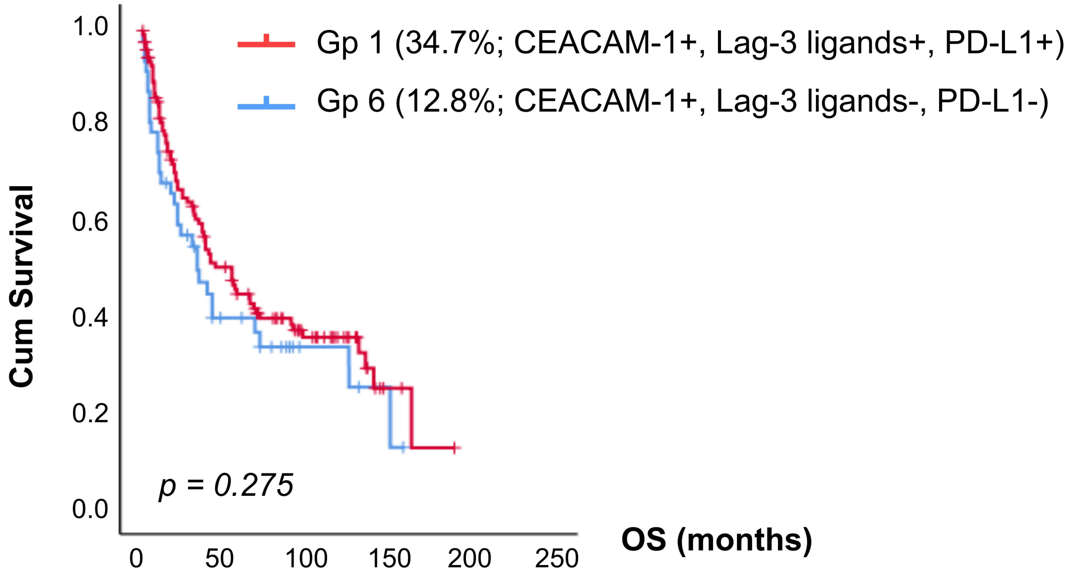

Supplementary Figure S2. Association of triple positive group and other combination group with survival

Gp 1 versus Gp5 (upper) or versus Gp6 (lower), which are mentioned in Figure 1d, respectively.
