# Supplementary material for: Combined inhibition of PD-1/PD-L1, Lag-3, and Tim-3 axes augments antitumor immunity in gastric cancer–T cell coculture models
Source: Gastric Cancer. 2021 Feb 20;24(3):611–23. doi: 10.1007/s10120-020-01151-8 (PMC8065004; doi:10.1007/s10120-020-01151-8)
Supplement: Supplementary file 3 — Supplementary file3 (PDF 7362kb) [file 10120_2020_1151_MOESM3_ESM.pdf]

Supplementary Figure S3

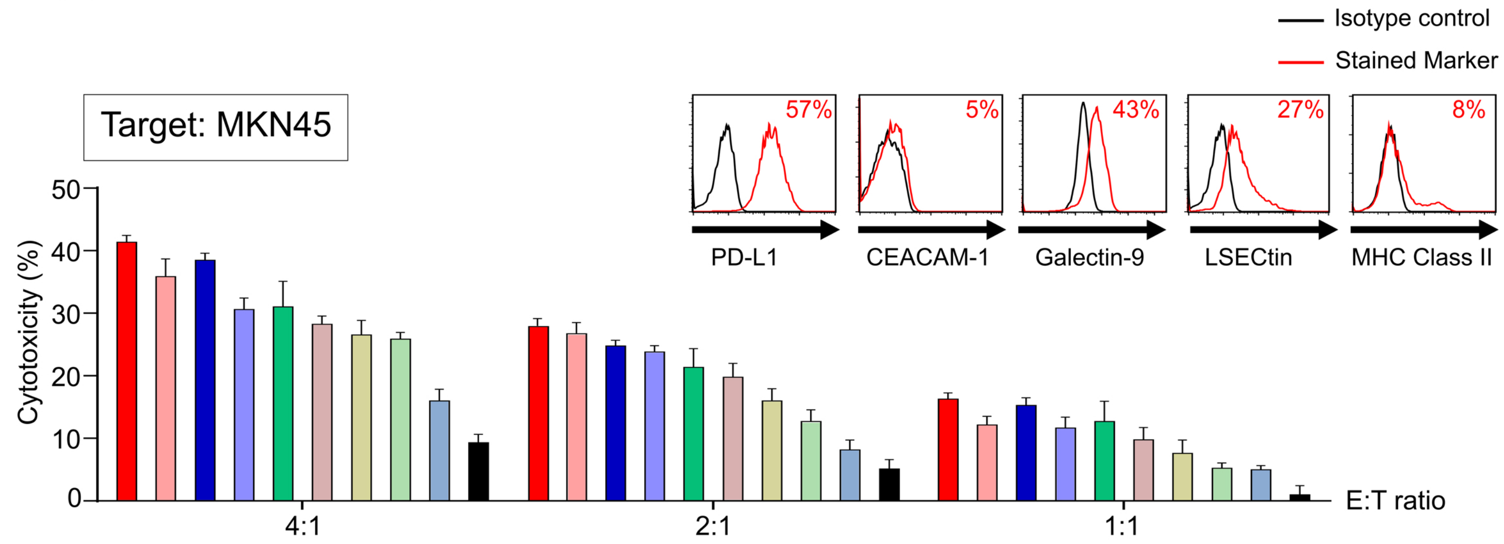

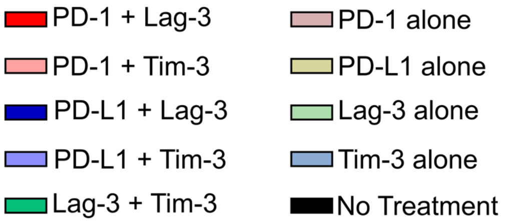

Single vs No Treatment	4:1	2:1	1:1
PD-1	****	****	***
PD-L1	****	***	*
Lag-3	****	**	ns
Tim-3	*	ns	ns

Dual vs Single	4:1	2:1	1:1
PD-1+Lag-3 vs PD-1	***	**	*
PD-1+Lag-3 vs Lag-3	****	****	****
PD-1+Tim-3 vs PD-1	*	*	ns
PD-1+Tim-3 vs Tim-3	****	****	*

Dual vs Single	4:1	2:1	1:1
PD-L1+Lag-3 vs PD-L1	****	**	**
PD-L1+Lag-3 vs Lag-3	****	****	***
PD-L1+Tim-3 vs PD-L1	ns	*	ns
PD-L1+Tim-3 vs Tim-3	****	****	*

Dual vs Single	4:1	2:1	1:1
Lag-3+Tim-3 vs Lag-3	ns	*	ns
Lag-3+Tim-3 vs Tim-3	***	**	ns

Dual vs Dual	4:1	2:1	1:1
PD-1+Lag-3 vs PD-1+Tim-3	ns	ns	ns
PD-1+Lag-3 vs PD-L1+Lag-3	ns	ns	ns
PD-1+Lag-3 vs PD-L1+Tim-3	**	ns	ns
PD-1+Lag-3 vs Lag-3+Tim-3	*	ns	ns
PD-1+Tim-3 vs PD-L1+Lag-3	ns	ns	ns

Dual vs Dual	4:1	2:1	1:1
PD-1+Tim-3 vs PD-L1+Tim-3	ns	ns	ns
PD-1+Tim-3 vs Lag-3+Tim-3	ns	ns	ns
PD-L1+Lag-3 vs PD-L1+Tim-3	ns	ns	ns
PD-L1+Lag-3 vs Lag-3+Tim-3	ns	ns	ns
PD-L1+Tim-3 vs Lag-3+Tim-3	ns	ns	ns

Supplementary Figure S3. Additive effect of different ICI combinations against MKN45

Cytotoxic activity of CTL clones were assessed in different dual ICI treatment settings.

Representative histograms of each inhibitory ligand expression are shown (top right).

Comparison between combinations were analyzed and presented in table (bottom).
