# Supplementary material for: Combined inhibition of PD-1/PD-L1, Lag-3, and Tim-3 axes augments antitumor immunity in gastric cancer–T cell coculture models
Source: Gastric Cancer. 2021 Feb 20;24(3):611–23. doi: 10.1007/s10120-020-01151-8 (PMC8065004; doi:10.1007/s10120-020-01151-8)
Supplement: Supplementary file 4 — Supplementary file4 (PDF 7562kb) [file 10120_2020_1151_MOESM4_ESM.pdf]

Supplementary Figure S4

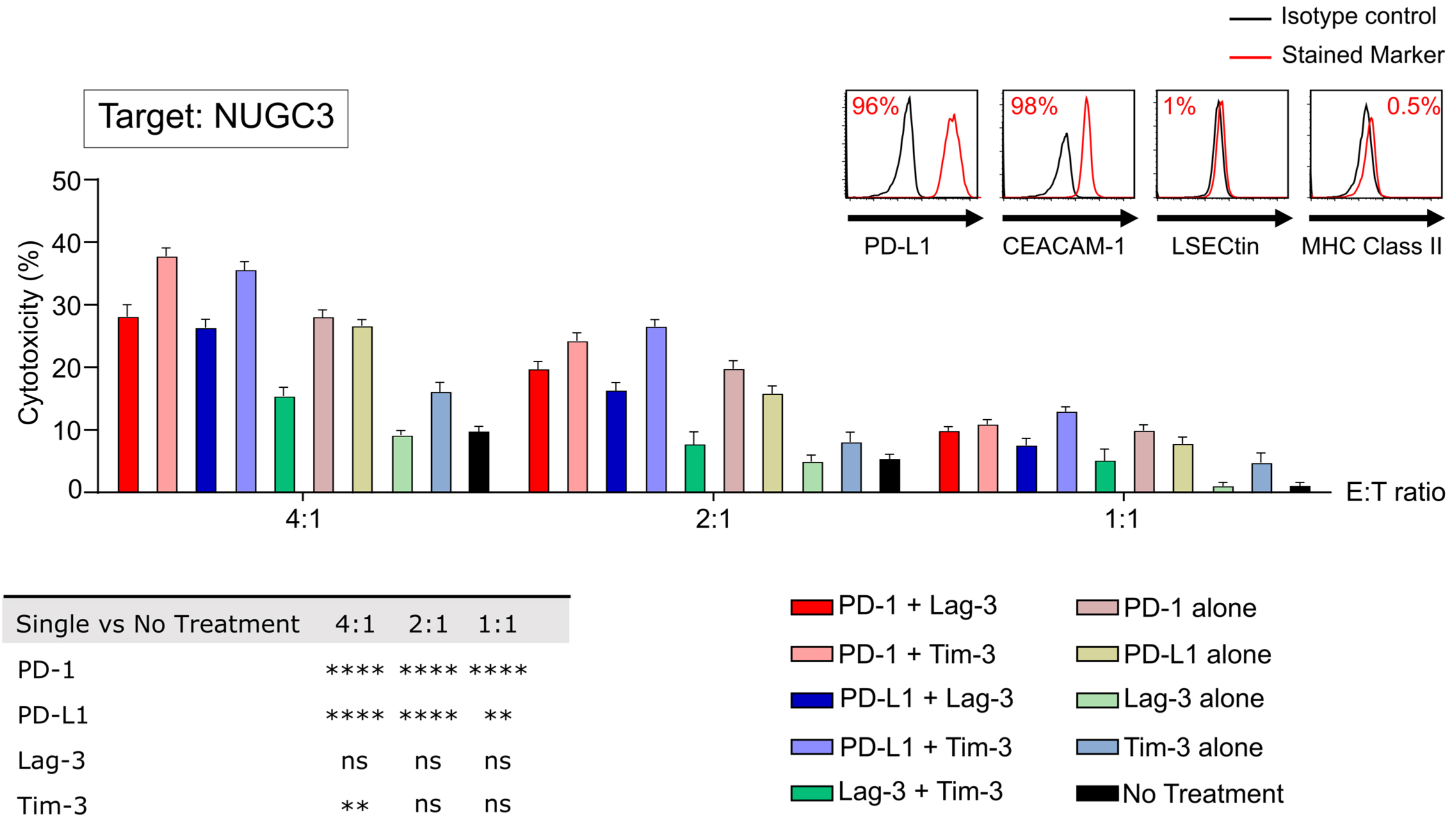

Single vs No Treatment	4:1	2:1	1:1
PD-1	****	****	****
PD-L1	****	****	**
Lag-3	ns	ns	ns
Tim-3	**	ns	ns

Dual vs Single	4:1	2:1	1:1
PD-1+Lag-3 vs PD-1	ns	ns	ns
PD-1+Lag-3 vs Lag-3	****	****	****
PD-1+Tim-3 vs PD-1	****	*	ns
PD-1+Tim-3 vs Tim-3	****	****	*
PD-L1+Lag-3 vs PD-L1	ns	ns	ns
PD-L1+Lag-3 vs Lag-3	****	****	***
PD-L1+Tim-3 vs PD-L1	***	****	*
PD-L1+Tim-3 vs Tim-3	****	****	*
Lag-3+Tim-3 vs Lag-3	ns	**	ns
Lag-3+Tim-3 vs Tim-3	ns	ns	ns

Dual vs Dual	4:1	2:1	1:1
PD-1+Lag-3 vs PD-1+Tim-3	***	ns	ns
PD-1+Lag-3 vs PD-L1+Lag-3	ns	ns	ns
PD-1+Lag-3 vs PD-L1+Tim-3	**	*	ns
PD-1+Lag-3 vs Lag-3+Tim-3	****	****	ns
PD-1+Tim-3 vs PD-L1+Lag-3	****	**	ns
PD-1+Tim-3 vs PD-L1+Tim-3	ns	ns	ns
PD-1+Tim-3 vs Lag-3+Tim-3	****	****	*
PD-L1+Lag-3 vs PD-L1+Tim-3	***	****	ns
PD-L1+Lag-3 vs Lag-3+Tim-3	****	***	ns
PD-L1+Tim-3 vs Lag-3+Tim-3	****	****	**

Supplementary Figure S4. Additive effect of different ICI combinations against NUGC3

Cytotoxic activity of CTL clones were assessed in different dual ICI treatment settings. Representative histograms of each inhibitory ligand expression are shown (top right). Comparison between combinations were analyzed and presented in table (bottom).
